# XPO1 target occupancy measurements confirm the selinexor recommended phase 2 dose

**DOI:** 10.18632/oncotarget.22801

**Published:** 2017-11-30

**Authors:** Marsha L. Crochiere, Stefan Hannus, Kerrin Hansen, Frank Becker, Erkan Baloglu, Margaret Lee, Michael Kauffman, Sharon Shacham, Yosef Landesman

**Affiliations:** ^1^ Karyopharm Therapeutics, Inc, Newton, MA 02459, USA; ^2^ Intana Bioscience GmbH, D-82152 Planegg/Martinsried, Munich, Germany

**Keywords:** selinexor, export, occupancy, cancer, dose

## Abstract

XPO1 (exportin 1) is the main nuclear export protein with over 200 different protein cargos. XPO1 is overexpressed in tumor cells and high levels are correlated with poor prognosis. Selective Inhibitor of Nuclear Export (SINE) compounds block nuclear export by inhibiting XPO1. The first SINE compound, selinexor, shows promising anti-cancer activity across hematological and solid tumors in Phase 2 and 3 clinical trials. The 2^nd^ generation SINE compound KPT-8602 is being evaluated as an anti-cancer agent in a Phase 1 clinical trial. To predict patient response to treatment and confirm the selinexor recommended phase 2 dose (RP2D), an assay based on fluorescence cross correlation spectroscopy that measures XPO1 occupancy in cancer cells was developed. Studies comparing cytotoxicity and XPO1 occupancy in cell lines treated with selinexor or KPT-8602 indicated that XPO1 occupancy by both compounds could reach saturation regardless of drug sensitivity. However, higher levels of XPO1 protein correlated with lower sensitivity to SINE compound cytotoxicity. *In vivo* mouse studies showed XPO1 occupancy could be measured in tumors and was dose-dependent, with >90% target saturation at 10 mg/kg (∼50 mg flat dose in humans). Drug-target occupancy was measured in a dose-response time course and full occupancy occurred by 6 hours at all doses. The duration of occupancy was dose-dependent, where 10-15 mg/kg in mice (∼ 50-75 mg human flat dose) was necessary to maintain XPO1 occupancy up to 48 hours post-dose. These findings confirm the selinexor RP2D of 60 mg for achieving target occupancy and inhibition up to 48 hours.

## INTRODUCTION

Target occupancy measurements quantify the amount of target bound to and possibly inhibited by the candidate compound. Ideally those measurements are carried out under physiological conditions to ensure predictability for subsequent steps in drug discovery and development. Assessing target occupancy is of critical importance to deepen the understanding of a biological target to human disease and to reconfirm the mode of action of a candidate drug. In later stages of drug development determination of target occupancy supports dose regimen and validation of biomarkers.

Identifying the optimal dose regimen for an anti-cancer drug to be efficacious is essential for ensuring patient safety while minimizing the occurrence of undesirable side effects. Clinical trials often compare many dose levels and treatment schedules to identify the regimen that results in the maximally tolerated dose for utilization in patient therapy [1], which may be more drug than is necessary to be the most efficacious. If more drug is administered than required to perform its function, the likelihood that the excess drug will affect pathways and processes which it was not intended to target increase, resulting in off-target effects [2]. Therefore, it is of value to determine the least amount of drug that is necessary to be the most beneficial for the patient. Measuring target occupancy in cells, xenograft tumors, surrogate tissue, and patient biopsy samples allows finding the minimal required quantity and optimal dosing scheme for a drug [3]. However, developing assays to quantify target occupancy for compounds can prove quite challenging [4, 5]. In addition, the *in vivo* conditions need to be maintained throughout the process of sample preparation and measurement to record the occupancy state at the time of sample collection. As such, sample preparation should be fast to retain the physiological conditions.

Selinexor is currently being evaluated in Phase 2 and 3 clinical trials for the treatment of cancer (clinicaltrials.gov). Selinexor, a Selective Inhibitor of Nuclear Export (SINE) compound, exerts its anti-cancer effect by binding to and inhibiting exportin 1 (XPO1), which is the main nuclear export protein for hundreds of cargoes including tumor suppressor proteins (TSPs) and growth regulatory proteins [6]. Selinexor treatment causes cell cycle arrest resulting in apoptosis and cell death in a variety of solid and hematologic malignancies [7]. KPT-8602 is a second-generation SINE compound also being evaluated for the treatment of several cancers in humans (NCT02649790; clinicaltrials.gov).

Selinexor has demonstrated efficacy in patients with heavily pretreated hematologic malignancies as well as with advanced or metastatic solid tumors. Preliminary safety and efficacy results for several of these studies can be found in selected publications [8–11]. Currently, the recommended phase 2 dose (RP2D) for selinexor is 60 mg twice weekly (BIW). While the RP2D was being identified empirically, an *in vitro* assay was developed to measure the amount of XPO1 occupied by selinexor in cancer cells to inform patient selection and dosing for selinexor therapy. That assay utilized biotinylated Leptomycin B (b-LMB) and Western blot analyses [12]. LMB is a natural product inhibitor of XPO1 and like SINE compounds binds to Cys528 in the cargo binding pocket of XPO1 [13]. Although LMB cannot be used clinically due to its high toxicity [14], the biotinylated form served as tool compound to measure the extent of XPO1 inhibition in cells *in vitro*, *ex vivo* and *in vivo*. Even though the b-LMB based assay successfully correlated occupancy of XPO1 to dosing level in PBMCs from mice and in cancer cells in culture, it could only measure the drug-target interaction in viable cells, which was not a feasible source of biopsy material from patients [12].

To overcome this limitation, a novel, fluorescence cross correlation spectroscopy (FCCS) based assay was developed with the goal of measuring target occupancy from non-viable patient biopsy samples. Fluorescence correlation spectroscopy (FCS) is a high-resolution spatial and temporal analysis of extremely low concentrated molecules. The parameter of primary interest is fluctuation of intensity caused by the diffusion of fluorescent molecules through a microscopic open detection volume in solution. This illuminated spot is generated by laser light, focused to its diffraction limit in combination with confocal optics. Recording the photons emitted from fluorescent molecules passing through the illuminated spot results in a fluctuation trace from which concentration of particles, molecular brightness and diffusion times of fast and slow particles can be calculated. FCCS allows simultaneous observation of two different species of fluorescent molecules by superimposing the detection spot with laser of 2 different wavelengths [15]. By this method, concentration and binding state of labeled molecules can be precisely accessed, in addition complex formation of two different fluorescent molecules can be recognized by codiffusion of both molecules through the detection spot and are scored as cross correlating particles.

Here the FCCS based target occupancy measurements were successfully employed to quantify the effects of selinexor treatment on the amount of XPO1 protein detected in cells pre- and post-treatment, to compare the occupancy of XPO1 by selinexor and KPT-8602 in cell lines with varying sensitivity to SINE compounds, to test the impact of freezing samples on the outcome of the assay, to evaluate PBMCs as a possible patient surrogate sample for the assay, and to test whether occupancy could be measured in tumor lysates from mice dosed with selinexor *in vivo* to mimic patient biopsy material. The results of this study indicate that the FCCS assay could indeed be utilized to measure occupancy from tumor tissue as well as confirm the selinexor RP2D of 60 mg flat dose administered BIW.

## RESULTS

### A novel FCCS assay quantifies target occupancy by selinexor and KPT-8602 in lysates from cancer cells

Precise quantification of target occupancy in relevant cancer cell lines and surrogate tissue is ideal information for the design of the drug dosing regimen in clinical studies for improved efficacy and reduced toxicity profile. To monitor the performance of candidate compounds in clinical studies, malignant tumor cells from a treated patient is the relevant test material. However, provision of sufficient amounts of biopsy material is challenging and requires freezing of samples to maintain the molecular status at the time of sample collection. A previously developed assay based on b-LMB failed to measure target occupancy by SINE compounds in frozen samples. To overcome this limitation, a novel test format based on FCCS was established. For this, cells treated *in vitro* or tissue samples from treated animals were processed to a homogeneous solution comprising all soluble cellular components including free and SINE compound-occupied XPO1 target proteins. Upon addition of labeled anti XPO1 specific antibodies (XPO1 AB_488_) and a LMB-Cy5 probe all unoccupied XPO1 target molecules were allowed to form trimeric complexes comprising both fluorophores. XPO1 target proteins that were pre-occupied with selinexor or KPT-8602 were recognized by the specific antibody, XPO1 AB_488_, but could not bind LMB-Cy5 and migrated similarly to the free XPO1 antibody and the LMB probe as single labeled molecules. By this assay, cross correlating particles precisely quantify the concentration of unoccupied target protein in the sample and allow quantifying precisely the degree of XPO1 target occupancy.

**Figure 1:**
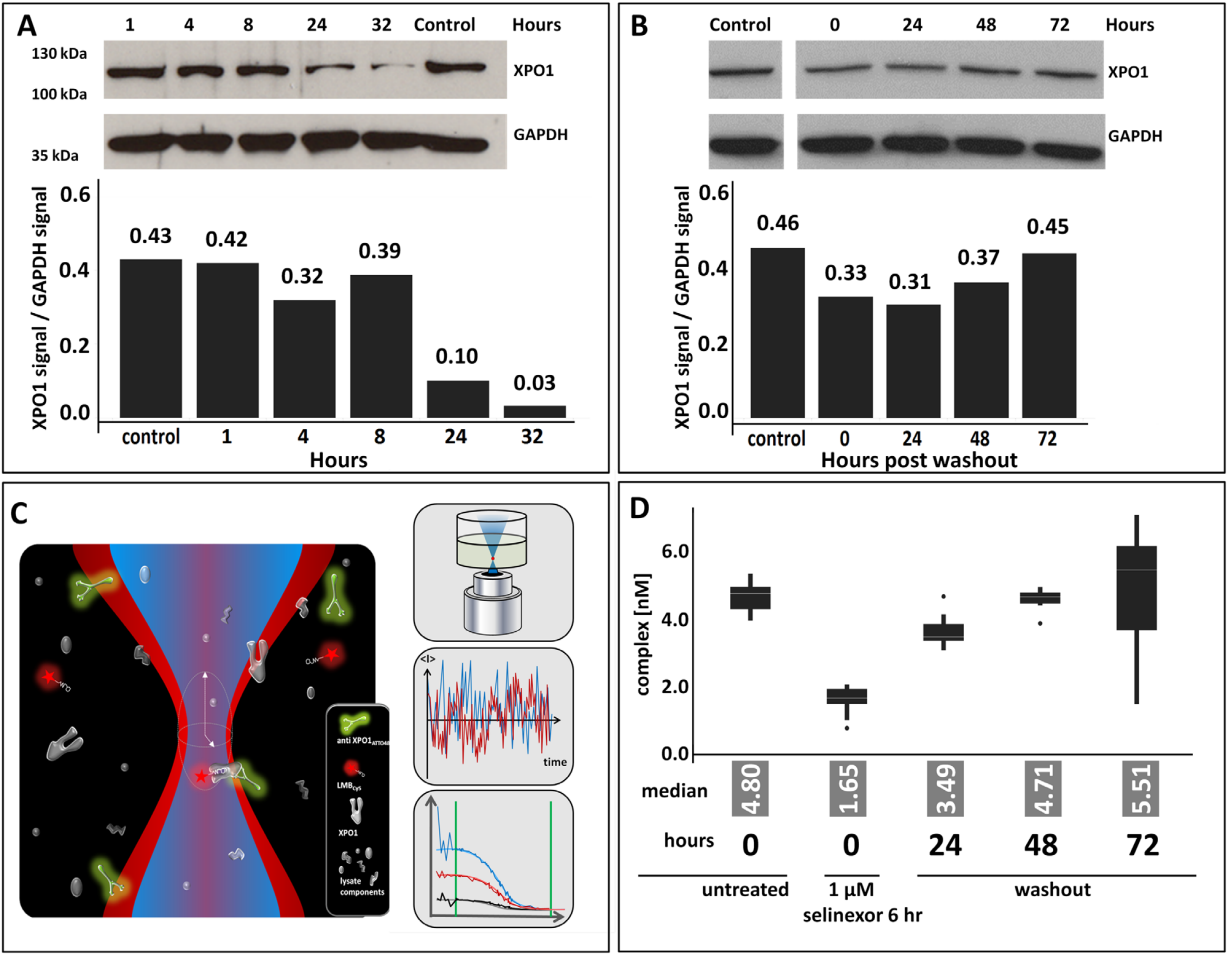
Selinexor treatment reduces expression of and occupies XPO1 **(A)** Z138 cells were treated with 1 uM selinexor continuously, harvested after 1, 4, 8, 24 and 32 hours and lysates were immunoblotted for XPO1. Expression of XPO1 at each time point was plotted by calculating the densitometric amount of XPO1 signal relative to GAPDH. **(B)** Z138 cells were treated with 1 uM selinexor for 6 hours, the drug was removed (washout), cells were harvested at 0, 24, 48, and 72 hours post washout then lysates were immunoblotted for XPO1. Expression of XPO1 at each time point was plotted by calculating the densitometric signal of XPO1 relative to GAPDH. **(C)** FCCS simultaneously measures fluctuations in 2 different fluorescent molecules diffusing through a microscopic detection spot. Interacting particles are scored by codiffusion and information is extracted on particle concentration, size, binding state, and molecular brightness. In the absence of selinexor, complexes of XPO1, the green labeled XPO1 antibody (aXPO1ABATTO488) and the red labeled LMB (LMBDY647), are formed and indicate low target occupancy. XPO1 occupied by SINE compound cannot bind the labeled tracer, decreasing dually labeled particles. Loss of complex formation is a measure of target occupancy in cells and tumor samples. **(D)** FCCS measurements of XPO1 occupancy from Z138 cells that were either untreated, treated for 6 hours, or treated for 6 hours then had the drug washed out and harvested at 24, 48, and 72 hours. Lysates were incubated with fluorescence labeled LMB (LMB_647_) and ATTO488 labeled anti XPO1 antibody (XPO1 AB_488_) for 2 hours and complex formation was analyzed on a ConfoCor2 Correlation Spectroscope. The values for the absolute number of XPO1 complexes were plotted. The box plots are presented to visualize the key statistical measures [median (line within the box), quartiles (Q1 – lower edge of the box; Q3 upper edge of the box), upper and lower adjacent values (whiskers – upper: largest value that is equal or less than Q3+1.5 times the IQR (inerquartile range - size of the square) or lower: smallest value that is greater or equal to Q1-1.5^*^IQR) and outliers (dots – data outside the fence defined by the upper and lower adjacent values)].

Selinexor inhibits nuclear export by covalently binding to cysteine 528 in the cargo binding pocket of the target protein XPO1. This inhibition is apparent in cell lines *in vitro* within 20 minutes and mouse xenograft tumors *in vivo* within 2 hours following drug treatment. Upon selinexor binding to XPO1 a decrease in the steady state levels of XPO1 protein is induced [16]. This leads to cell cycle arrest and subsequent cancer cell death [17]. It was therefore necessary to evaluate initially how the duration of drug exposure affected XPO1 protein levels to then measure occupancy. Cells from the mantel cell lymphoma cell line Z138 were treated with 1 uM selinexor either continuously and harvested after 1, 4, 8, 24 and 32 hours or they were treated for 6 hours, the drug was then removed (washout) by changing the media, and harvested at 0, 24, 48, and 72 hours post-washout. Lysates immunoblotted for XPO1 showed that continuous exposure to selinexor resulted in a dramatic, sustained reduction of protein while transient exposure to selinexor resulted in a moderate yet prolonged reduction of XPO1 (Figure 1). With continuous exposure, at 32 hours of drug treatment there was a 93% reduction of XPO1 protein compared to the control (Figure 1A) whereas after 6 hours of exposure followed by drug wash out, at 24 hours (30 hours total incubation time) there was only a 33% reduction of XPO1 (Figure 1B). This confirmed that the presence of selinexor is required to sustain decreased XPO1 protein and informed the conditions necessary to optimally perform the FCCS assay.

Based on these observations, Z138 cells were treated for 6 hours, washed, incubated for 24, 48, and 72 hours post-washout (see Materials and Methods for details, Figure 1C and 1D) and processed to cellular lysates. Subsequently, lysates were incubated with labeled LMB and labeled anti-XPO1 and subjected to FCCS analysis (depicted in Figure 1C). The absolute number of complexes detected at each time point were plotted. In the absence of selinexor, a high number of complexes were detected (Figure 1D). After 6 hours of treatment with selinexor at time 0 hours post-washout, almost no complexes were detected, indicating that all XPO1 proteins were occupied by selinexor and could not bind the labeled LMB. Over time post-washout, the number of complexes detected increased indicating that either the binding of selinexor to XPO1 was reversible [reviewed in (13)], or that new XPO1 protein was translated and not enough free selinexor was present in the lysate to occupy the new protein. By 48 hours post washout, nearly all XPO1 was free of selinexor. These results suggest that drug exposure as brief as 6 hours is sufficient to completely occupy the target and that occupancy persists for up to 48 hours, and that either higher concentrations with brief exposure or lower concentrations with continuous exposure of drug is necessary to maintain durable drug-target interaction. This result also indicates that in order to accurately measure occupancy, the experiments should be performed after brief selinexor exposure so as not to interfere with the levels of protein available for drug binding.

### SINE compounds occupy XPO1 similarly in a variety of hematologic cell lines regardless of drug sensitivity

**Figure 2:**
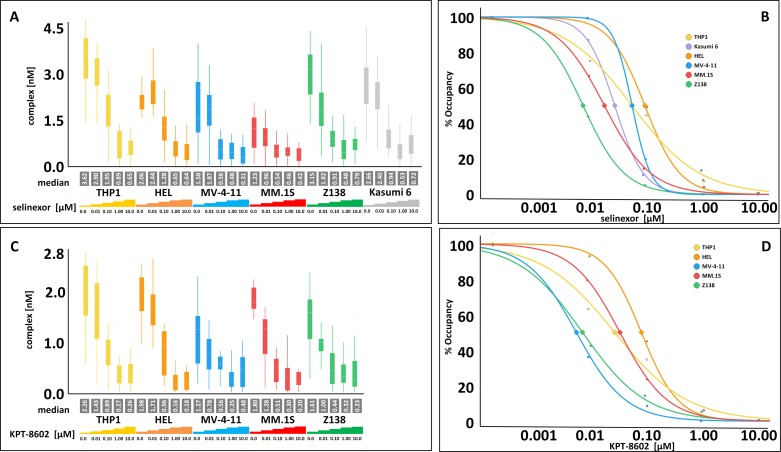
Selinexor and KPT-8602 dose-dependently occupy XPO1 in hematologic cell lines *in vitro* with no correlation to drug sensitivity **(A)** FCCS measurements of XPO1 occupancy from THP-1, HEL, MV-4-11, MM.1S, Z138, and Kasumi-6 cell lines grown to confluency and treated with 0, 0.01, 0.1, 1, and 10 μM selinexor for 4 hours. Cells were washed, harvested and lysed using PBS-Tween buffer and incubated with fluorescence labeled LMB (LMB_647_) at a concentration of approximately 25 nM and ATTO488 labeled anti XPO1 antibody (XPO1 AB_488_) for 2 hours and complex formation was analyzed on a ConfoCor2 Correlation Spectroscope. **(B)** The normalized (untreated = 100%) values for the XPO1 complexes were plotted for selinexor. **(C)** FCCS measurements of XPO1 occupancy from THP-1, HEL, MV-4-11, MM.1S, and Z138 cell lines grown to confluency and treated with 0, 0.01, 0.1, 1, and 10 μM KPT-8602 for 4 hours. Cells were washed, harvested and lysed using PBS-Tween buffer and incubated with fluorescence labeled LMB (LMB_647_) at a concentration of approximately 25 nM and ATTO488 labeled anti XPO1 antibody (XPO1 AB_488_) for 2 hours and complex formation was analyzed on a ConfoCor2 Correlation Spectroscope. **(D)** The normalized (untreated = 100%) values for the XPO1 complexes were plotted for KPT-8602. The resulting values were fitted to a logistic regression curve using the TIBCO Spotfire software package.

XPO1 occupancy was first compared between selinexor and KPT-8602, and then XPO1 occupancy measurements in resistant and sensitive cell lines were compared for each compound (Figure 2, Table 1, and see [12]). A panel of hematologic cell lines were treated with 0, 0.01, 0.1, 1, and 10 μM selinexor (Figure 2A) or KPT-8602 (Figure 2C) for 4 hours and then harvested for the FCCS assay. The cells ranged from SINE compound resistant (THP-1, Kasumi-6, and HEL) to sensitive (MV-4-11, MM.1S, and Z138). Curves were then generated for each cell line tested with either selinexor (Figure 2B) or KPT-8602 (Figure 2D) to extrapolate the concentration of drug required to occupy 50% and 90% of the XPO1 protein. The selinexor cytotoxicity IC_50_ values for the cell lines were reported previously [12] while the KPT-8602 cytotoxicity IC_50_ values, selinexor and KPT-8602 50% and 90% occupancy values, as well as the relative amount of XPO1 protein to GAPDH for THP-1, HEL, MV-4-11, MM.1S, and Z138 are presented in Table 1. Selinexor and KPT-8602 both dose-dependently occupied XPO1 in all cell lines tested. The occupancy values were similar for each compound suggesting there was no difference between the ability of selinexor or KPT-8602 to bind XPO1, and no correlation was observed between SINE compound sensitivity and occupancy value, consistent with the observation made with the previous occupancy assay [12]. Occupancy values for selinexor and KPT-8602 were similar for each cell line except for Kasumi-6 which was not tested with KPT-8602, or for MV-4-11, which was ∼10-fold more sensitive to selinexor than to KPT-8602 (Table 1). For selinexor, 50% occupancy occurred between 8.3 to 100 nM, while for KPT-8602, 50% occupancy occurred between 6.2 to 85.1 nM. For selinexor, 90% occupancy occurred between 52.5 to 1202.3 nM, while for KPT-8602, 90% occupancy occurred between 54.3 to 728.9 nM. However, cells which were more resistant (THP-1, HEL) tended to have higher levels of XPO1 protein than cells that were more sensitive (MV-4-11, MM.1S, Z138). These findings suggest that SINE compound resistance is conferred by mechanisms that occur downstream of drug-target interaction. Therefor the occupancy assay alone is not sufficient to inform patient selection for SINE compound therapy [12, 17]. The data further indicate that the relative amount of XPO1 protein inversely correlates with drug sensitivity.

**Table 1:** There is no correlation between drug sensitivity and occupancy for either selinexor or KPT-8602 in any cell line, but there is a correlation between drug sensitivity and relative XPO1 protein amounts

Cell line	KPT-8602 IC_50_ (nM)	Selinexor 50% occupancy (nM)	KPT-8602 50% occupancy (nM)	Selinexor 90% occupancy (nM)	KPT-8602 90% occupancy (nM)	XPO1 Protein (relative XPO1 / GAPDH)
THP-1	3380	58.9	28.8	1202.3	728.9	1.89
HEL	8667	100.0	85.1	478.6	433.3	1.21
MV-4-11	57	60.3	6.2	138.0	54.3	1.08
MM.1S	50	23.9	35.4	154.9	293.4	0.39
Z138	6	8.3	7.9	52.5	90.8	0.67

The values obtained for the hill factor and the 50% occupancy value were used to calculate the 90% occupancy values using GraphPad for selinexor and KPT-8602.

As a comparator to selinexor and KPT-8602, the FCCS occupancy assay was also performed on cell lines treated with 0, 0.01, 0.1, 1, and 10 μM LMB (Supplementary Figure 1). LMB exhibited very strong, nearly complete XPO1 occupancy in all cells lines (THP-1, HEL, MV-4-11, MM.1S, and Z138) at the lowest concentration tested (0.01 μM).

### The FCCS occupancy assay measures drug-target binding from frozen samples

**Figure 3:**
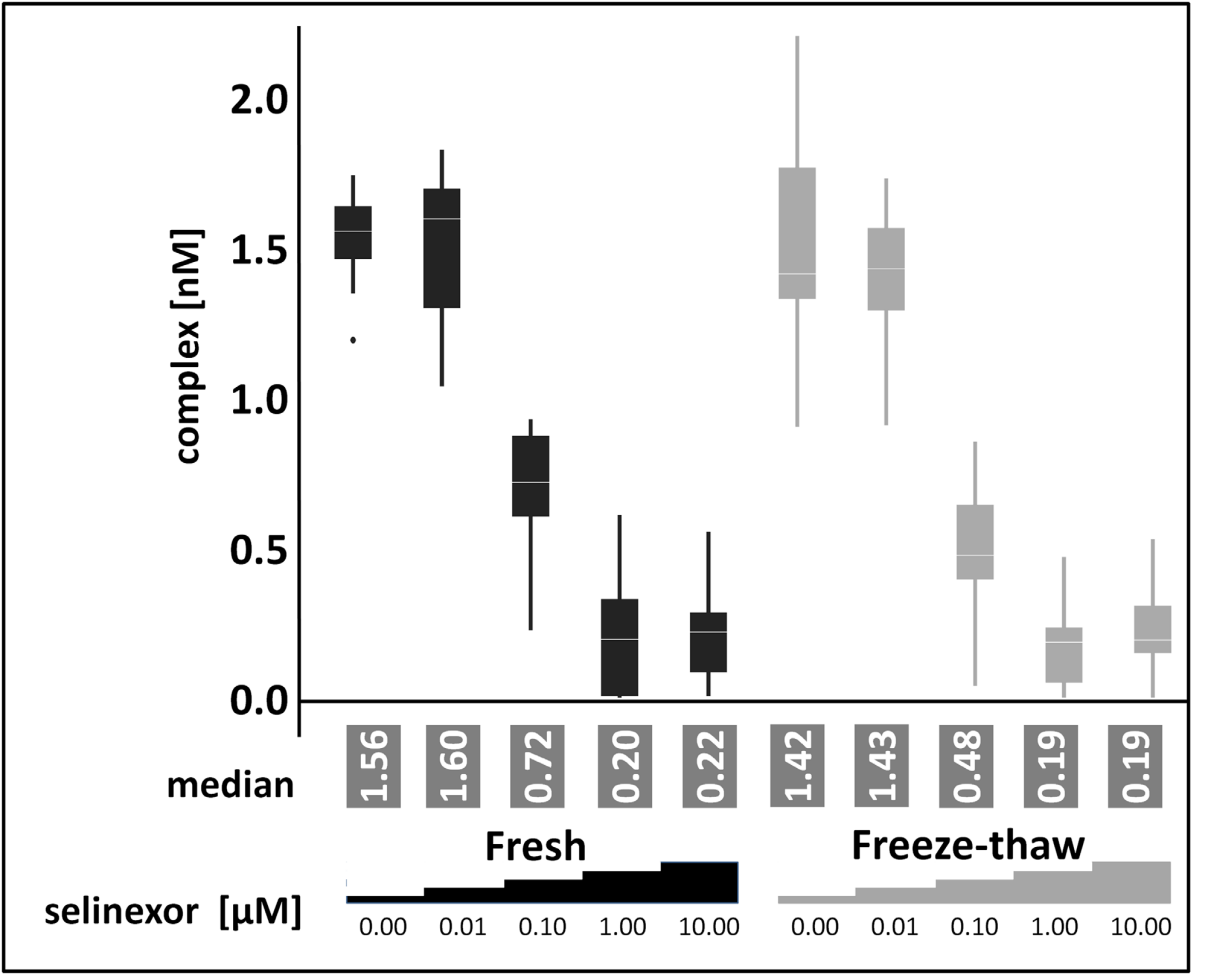
Freezing and thawing has no impact on target occupancy FCCS measurements of XPO1 occupancy from HEK293 cells treated for 4 hours with 0, 0.01, 0.1, 1, or 10 μM selinexor from lysates processed immediately or after freeze-thaw. Cell lysates were coincubated with fluorescence labeled LMB (LMB_647_) and ATTO488 labeled anti XPO1 antibody (XPO-1 AB_488_) for 2 hours and measured on a ConfoCor2 Correlation Spectroscope. The absolute values for the XPO1 complexes were plotted. The resulting values were fitted to a logistic regression curve using the TIBCO Spotfire software package.

After demonstrating the feasibility of FCCS based target occupancy measurements the next step was to test the approach on frozen samples. Freezing of samples is preferred because the amount of patient biopsy is limited and maintaining fresh biopsy material as well as preserving the *in vivo* drug-target interaction is technically challenging. To test the assay with an adherent cell line in addition to comparing fresh and frozen samples, HEK293 cells were treated in culture for 4 hours with 0, 0.01, 0.1, 1, or 10 μM selinexor. The cells were harvested and lysates were prepared. Half of the lysate was processed immediately for the FCCS assay (fresh, Figure 3), while the other half was frozen prior to preparation for the FCCS assay after thawing (freeze-thaw, Figure 3). The cross-correlation amplitude for both conditions was very similar at all selinexor concentrations. The percent of XPO1 occupied by labeled LMB and labeled antibody were comparable at each concentration of selinexor tested for both fresh and freeze-thaw conditions, with XPO1 occupancy reaching saturation (83.3% in fresh and 88.2% in freeze-thaw) at 1 μM (Table 2). To validate the approach for the intended application in clinical testing intact cells were subjected to freeze thaw cycles prior to lysate preparation and XPO1 occupancy levels were compared with unfrozen control cells. The measurement showed no difference between frozen cells, frozen lysates and fresh cells (data not shown). This result indicated that the assay could indeed be utilized for measuring occupancy from frozen samples.

**Table 2:** The percent of XPO1 occupied by labeled LMB and labeled antibody for each concentration of selinexor tested in HEK293 cells are similar in both conditions

	% Occupancy	
Selinexor [μM]	Fresh	Freeze - thaw
0	0.0	0.0
0.01	11.1	0.0
0.1	55.6	52.9
1	83.3	88.2
10	83.3	76.5

The values obtained for the hill factor and the IC_50_ value were calculated using GraphPad.

### Application of FCCS based target occupancy measurements as an accompanying analytic tool in the clinic

**Figure 4:**
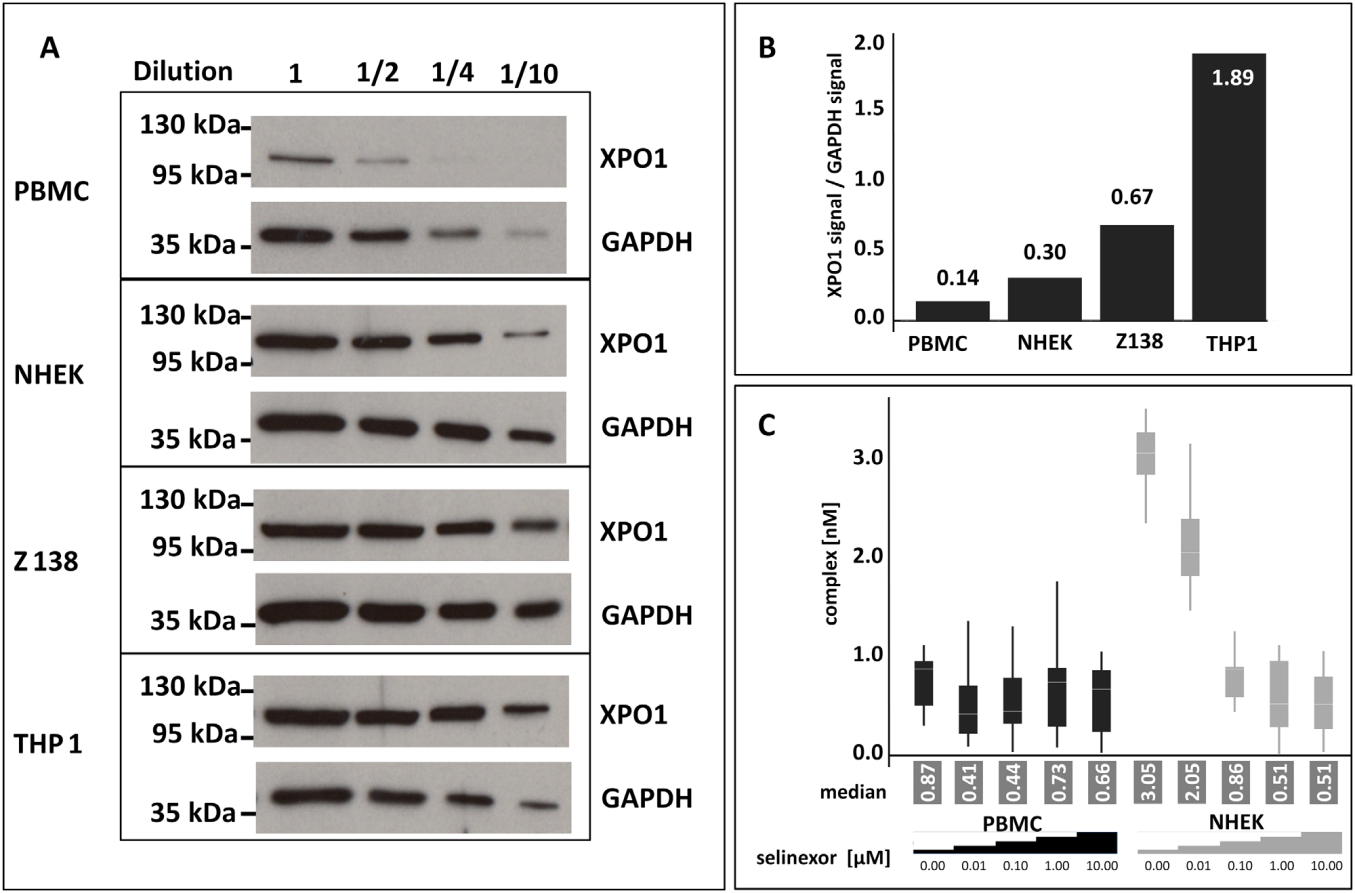
PBMCs are not adequate for measuring XPO1 occupancy by FCCS expression **(A)** Untreated PBMCs, NHEK, Z138, and THP-1 cells were harvested, lysed, and equivalent amounts of serially diluted lysates from each cell type were immunoblotted for XPO1. **(B)** Expression levels of XPO1for each cell type were calculated relative to their GAPDH signal. **(C)** FCCS measurements of XPO1 occupancy from PBMCs and NHEK treated with 0, 0.01, 0.1, 1, or 10 μM selinexor for 4 hours. Lysates were incubated with fluorescence labeled LMB (LMB_647_) and ATTO488 labeled anti XPO1 antibody (XPO1 AB_488_) for 2 hours and complex formation was analyzed on a ConfoCor2 Correlation Spectroscope. The absolute values for the XPO1 complexes were plotted. The resulting values were fitted to a logistic regression curve using the TIBCO Spotfire software package.

In the era of precision medicine and personalized healthcare physicians strive to provide patients with drugs that specifically seize the molecular cause of disease for better efficacy and tolerability. Relating target occupancy to the success of medication supports adjusting the therapeutic strategy to the patients’ needs and can be employed as analytical tool to rationalize the dosing and regimen of treatment. Since PBMCs are a less invasive sample to obtain to evaluate selinexor dosing (although not necessarily a biologically-relevant means to evaluate drug-target interaction occurring in the tumor), it was worth investigating whether the FCCS assay could also measure occupancy from PBMCs. PBMCs were isolated from healthy human donor whole blood. NHEK cells were tested as a normal cell comparator, and Z138 and THP-1 cells were included as controls (Figure 4). The cells were treated with 0, 0.01, 0.1, 1, or 10 μM selinexor for 4 hours. Equivalent amounts of serially-diluted lysates from each cell type were immunoblotted for XPO1 and found that the expression of XPO1 compared to GAPDH from PBMCs was very low (93% less XPO1 expressed than in THP-1 cells, Figure 4A, 4B). The occupancy assay was performed on these lysates and the low level of XPO1 protein in the PBMC cell lysate made it difficult to detect complex formation even in the absence of selinexor (Figure 4C). NHEK cells, however, had only 16% less XPO1 protein compared to THP-1 cells and XPO1 occupancy could be measured from these cell lysates with comparable values to those of Z138 cells at each concentration of selinexor tested (see Figure 2A). These results show that although the FCCS assay can measure XPO1 occupancy from frozen samples, PBMCs cannot be utilized as a sample source from patients for evaluating target engagement or dosing.

**Figure 5:**
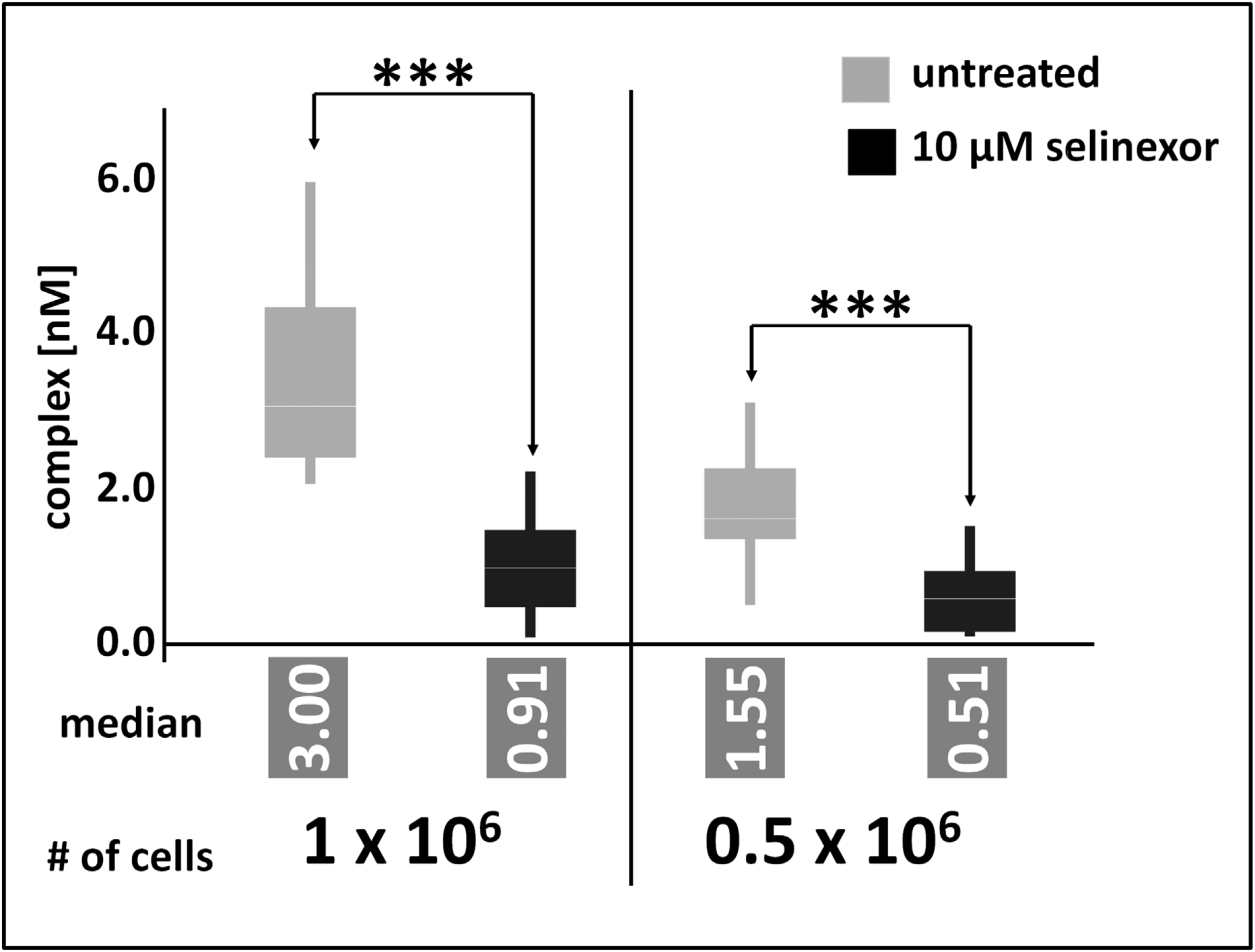
FCCS application as an analytical tool in clinical studies Z138 cells were counted and treated for 4 hours with 10μM selinexor, washed and directly processed to cellular lysates. Lysates were incubated with fluorescence labeled LMB (LMB_647_) and ATTO488 labeled anti XPO1 antibody (XPO1 AB_488_) for 2 hours and complex formation was analyzed on a ConfoCor2 Correlation Spectroscope. The absolute values for the XPO1 complexes were plotted. Measurements were done either 100 times for 8 seconds for 1 × 10^6^ cells or 200 times for 0.5 × 10^6^ cells. Differences between control and treated samples were significant based on the p-value determined with a standard t-Test using the excel statistics plugin, ^***^ indicates p-values obtained: 1 × 10^6^ cells, p = 2.69 × 10^-24^; 0.5 × 10^6^ cells, p = 4.8 × 10^-15^.

Because PBMC could not be used as surrogate cell population to measure XPO1 occupancy by selinexor due to low target expression, instead the minimal amount of tumor cells required for reliable occupancy measurements was defined. For previous measurements of *in vitro* cultivated cells, 2 × 10^7^ cells were processed to lysates and aliquots corresponding to 8 × 10^6^ cells were subjected to FCCS measurements. Here, the amount of Z138 cells was gradually reduced to identify the quantification limit of the FCCS approach. At the same time assay conditions were modified to enhance the sensitivity of the assays. Cells were treated for 4 hours with 10μM selinexor, washed and directly processed to cellular lysates. Aliquots were incubated with elevated amounts of labeled antibody and tracer and incubation time was prolonged to 2 hours. Finally, the detection volume of the instrument was enlarged by changing the pinhole setting for the blue and the red laser lines from 70 μm and 90 μm to 100 μm und 120 μm, respectively. Under these conditions FCCS measurements could detect a significant difference of complex formation in cellular lysates prepared from 0.5 × 10^6^ cells in response to selinexor treatment. In absolute numbers, the measured lysate corresponded to as little as 0.25 × 10^6^ treated cells (Figure 5) and resulted in a median of 0.5 nM complex (XPO1 bound to labeled antibody and LMB-Cy5) whereas the detected amount of complex from untreated cells was significantly higher (1.55 nM). Lysates prepared from 1 × 10^6^ cells were measured accordingly and resulted in 0.5 nM complex for the treated cells and 3 nM complex for the untreated cells. These data show that albeit PBMC could not be analyzed by FCCS the approach has the potential for application as an analytical tool in clinical studies.

### FCCS measurement of selinexor occupancy of XPO1 *in vivo* confirms the RP2D

**Figure 6:**
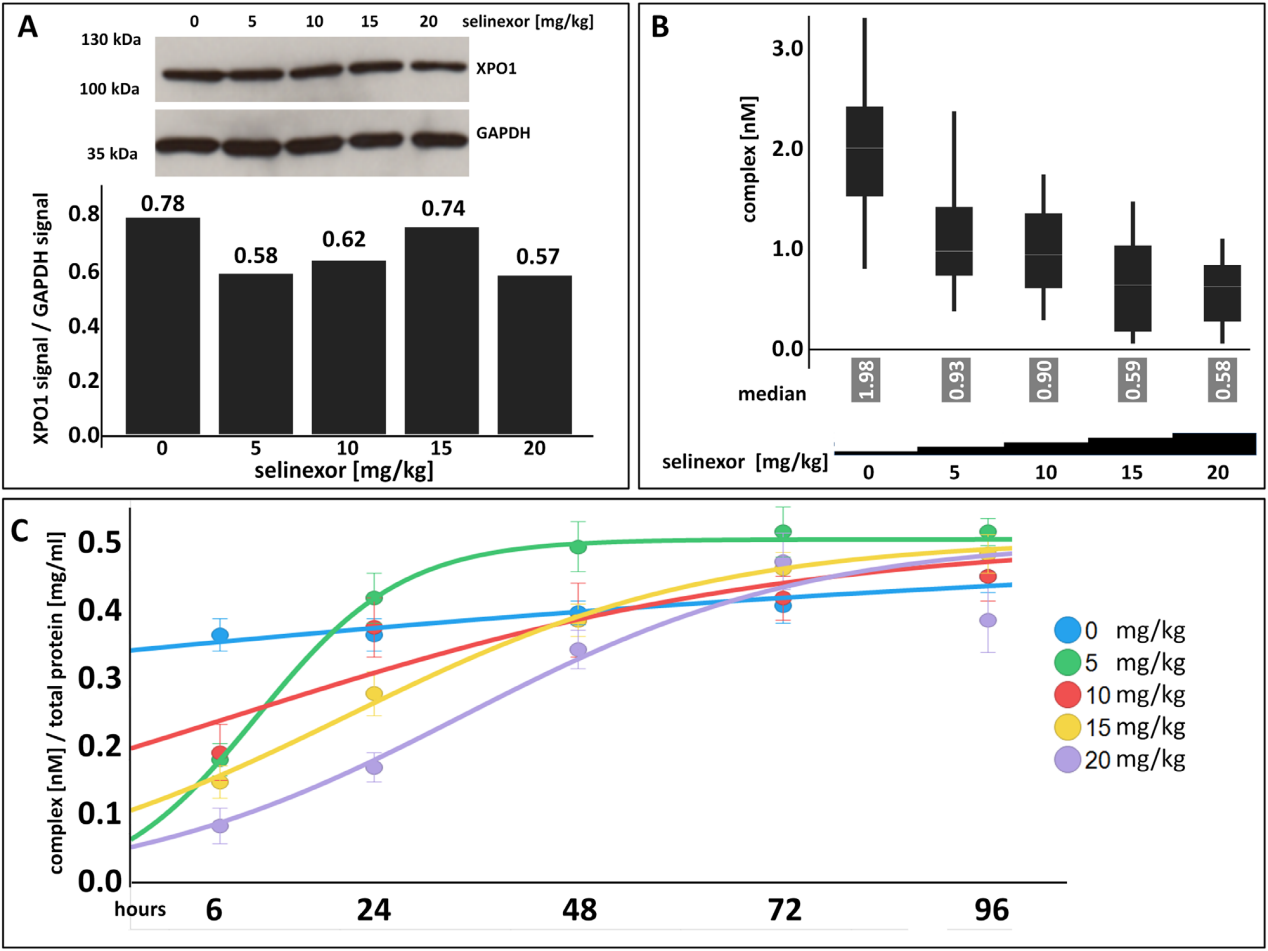
XPO1 target occupancy can be measured in tumors with a dose-dependent effect of selinexor on complex formation where target saturation occurs at ∼10 mg/kg selinexor and confirms the selinexor RP2D **(A)** XPO1 protein levels were detected from tumors harvested from mice bearing Z138 tumor xenografts that were orally administered a single dose of 0, 5, 10, 15, or 20 mg/kg selinexor and harvested at 6 hours post single dose. Expression of XPO1 was plotted by calculating the densitometric amount of XPO1 to GAPDH signal. **(B)** FCCS measurements of XPO1 occupancy from tumor lysates incubated with fluorescence labeled LMB (LMB_647_) and ATTO488 labeled anti XPO1 antibody (XPO-1 AB_488_) for 2 hours and complex formation was measured on a ConfoCor2 Correlation Spectroscope. The absolute values for the XPO1 complexes were plotted. **(C)** FCCS measurements of XPO1 occupancy from Z138 tumors from xenografted mice that were orally administered a single dose of 0, 5, 10, 15, or 20 mg/kg selinexor and harvested at 6, 24, 48, 72, and 96 hours post single dose. Samples were lysed and incubated with fluorescence labeled LMB (LMB_647_) and ATTO488 labeled anti XPO1 antibody (XPO-1 AB_488_) for 2 hours and complex formation was measured on a ConfoCor2 Correlation Spectroscope. Due to the variation in the size of the tumors, the concentration of dually labeled particles was normalized to total protein concentration. The resulting values were fitted to a logistic regression curve using the TIBCO Spotfire software package.

Tumor biopsies from patients are the most relevant sample to measure target occupancy. The assay reveals whether candidate drugs enter the relevant tissue, the time until compounds occupy the target and how long occupancy is maintained after compound withdrawal. Target occupancy measurements thus support understanding of important pharmaco-kinetic parameters. To test whether the FCCS assay could be utilized to uncover these features in patient samples, occupancy measurements were tested in frozen tumor tissue of mice implanted with Z138 cells. After tumors had grown to approximately 400 mm^3^, a single dose of either 0, 5, 10, 15, or 20 mg/kg selinexor was administered by oral gavage (Figure 6A, 6B). Tumors were harvested 6 hours post-dose and lysates were prepared for immunoblot analysis and the FCCS assay. After 6 hours post-dose, there was a slight decrease in the amount of XPO1 protein with increasing drug concentration relative to the untreated control (Figure 6A). FCCS measurement of the complexes showed a dose-dependent decrease in the number of complexes with increasing concentration of selinexor and saturated at 10 mg/kg (Figure 6B). Fifty% occupancy was reached at ∼3.3 mg/kg and 90% occupancy was reached at ∼13 mg/kg. In mice, XPO1 occupancy saturation at ∼10 mg/kg (∼50 mg flat dose in humans) is consistent a dose that shows anti-cancer activity in patients with heavily pretreated hematologic and solid tumors [8, 9, 18].

To determine the *in vivo* duration of drug-target interaction post administration of a single dose of selinexor, a dose-response time course in Z138 xenografted mice was performed (Figure 6C). Mice were inoculated with Z138 cells and allowed to grow tumors. Mice bearing tumors were then orally administered a single dose of 0, 5, 10, 15, or 20 mg/kg selinexor. Tumors from mice at each dose level were harvested at 6, 24, 48, 72, and 96 hours post-dose. Measurements by FCCS showed a dose- and time-dependent correlation to the number of complexes detected (Figure 6C). XPO1 proteins were dose-dependently occupied by selinexor at 6 hours post-treatment and the number of complexes detected increased dose-proportionately with time. Similar to the *in vitro* washout experiment (Figure 1D), 6 hours of treatment was sufficient to produce a drug-target interaction that was measurable in tumors *in vivo*. Tumor and body weights were recorded, and tumor volumes decreased slightly by 96 hours at doses of 15 and 20 mg/kg (data not shown), whereas body weights did not change significantly at any dose with time (data not shown). These data show that maximal occupancy occurred at 6 hours for all doses tested and persisted until 48 hours at 10 to 20 mg/kg (where 10 to 20 mg/kg in mice is approximately equivalent to 50 to 100 mg flat dose in humans). This duration of occupancy in animals supports the RP2D dosing regimen of 60 mg twice weekly on days 1 and 3. Such dosing regimen maintains steady state levels of drug-target interaction during the first half of each treatment week and washout / clearance of drug in the second half of the week, resulting in cancer cell growth inhibition / death while allowing recovery of normal tissue [19].

## DISCUSSION

Assays to measure target occupancy have been developed to verify drug-target engagement for a variety of indications using many different methods such as microscopic detection of fluorescently labeled probes [20, 21], positron emission tomography of tracers [22, 23], or flow cytometry [reviewed in 3, 4]. Fluorescently labeled molecules can be used as probes to measure target occupancy for the Bruton tyrosine kinase inhibitor ibrutinib in PBMCs [21], or when combined with fluorescent polarized microscopy to quantitate drug-target engagement in single cells [24]. FCCS was recently used to set up homogeneous binding assays to measure receptor-ligand binding affinities for G protein-coupled receptors, which is particularly challenging to accomplish with this family of receptors [20]. The approach can be applied in cellular lysates and requires only minimal amounts of test substance which substantially facilitates assay development and accelerates sample preparation. Measuring target occupancy is increasingly considered a critical step in drug discovery and clinical drug development, but imposes a set of experimental difficulties- namely, lengthy sample preparation will alter the cellular conditions and distort the results of occupancy measurements. Investigation of drug target interaction *in vivo* is close to impossible without employing modified compounds, but label-free experiments are feasible only *in vitro*. FCCS was chosen to directly measure XPO1 occupancy by SINE compounds in cells and tumors following treatment because analysis is carried out under conditions that approximate the *in vivo* situation and both drug and target can be measured label-free with *in vitro* accuracy.

In this study, the FCCS occupancy assay was used to compare the interaction of XPO1 with two different clinical stage SINE compounds, selinexor and KPT-8602. The assay also compared XPO1 occupancy in cells with different sensitivity to the SINE compounds to simulate patient stratification for therapy. Lastly the assay confirmed the appropriate amount of drug to dose patients to minimize off-target effects by measuring XPO1 occupancy in tumors from mice treated with selinexor. Although the FCCS assay did not detect differences in target interaction between selinexor or KPT-8602 nor differences in target binding in drug-sensitive versus resistant cells, it did confirm the selinexor RP2D dose of 60 mg and suggest that BIW doses (Monday and Wednesday), achieve ∼ 48 hours of target inhibition.

An assay that was based on affinity purification of XPO1 bound to b-LMB was successfully employed previously to measure target occupancy by SINE compounds (10). The multi-step approach was successful because SINE compounds are slowly reversible small molecule inhibitors that covalently bind to the cysteine 528 in the cargo binding pocket of XPO1 [reviewed in 13]. Accordingly, complexes formed between selinexor and the 2^nd^ generation SINE compound KPT-8602 will not dissociate upon cell lysis, dilution and purification and yield reliable target occupancy results. Of note, XPO1 occupancy measurements based on the b-LMB approach corresponded well to the data obtained with the FCCS methodology. Moreover, XPO1 occupancy measurements in PBMC cells that were chosen as surrogate patient material for easier accessibility failed with FCCS but were successfully carried out using b-LMB and Western blotting. This can be explained by the conceptual difference between both experimental approaches. FCCS measurements record diffusion events in real time and rare events are difficult to assess whereas the Western blot based approach used in the previous assay involved a purification step of XPO1 bound to the b-LMB which was then detected by Simple Western [see (12) for details] and thereby amplified the signal. However, the Western blot based approach failed to measure occupancy in previously frozen samples. In view of the difficulty to provide patient tissue in sufficient amounts and the experimental set up required to perform target occupancy measurements, sample storage is inevitable. PBMCs have been shown to express XPO1 at very low levels, whereas relevant cancer cell lines express the target at concentrations that allow FCCS analysis. In order to validate FCCS as a clinical analytical tool, the approach has been optimized for analysis of minimal cell numbers. Currently, a single measurement can be carried out with lysates corresponding to 0.25 × 10^6^ Z138 cells. Future experiments will show whether this sensitivity is sufficient to allow reliable target occupancy measurements in patient tissue samples.

Reduction of XPO1 protein is typically used as a biomarker to indicate target engagement by selinexor in preclinical and clinical studies [18, 25, 26]. To understand the dynamics between protein and drug engagement and inform the conditions necessary for the FCCS assay, XPO1 protein levels were evaluated in Z138 cells treated with selinexor either continuously or after drug removal. Continuous treatment was required to maintain reduced XPO1 protein levels; XPO1 protein was reduced by 26% as early as 4 hours of treatment and by 77% at 24 hours. Cells treated with selinexor for 6 hours had XPO1 protein reduced by 28% but after drug washout XPO1 protein was only reduced by 33% at 24 hours post-wash out (equivalent to 30 hours post-treatment) compared to 93% reduction after 32 hours of continuous treatment. XPO1 expression was nearly fully restored by 72 hours, indicating that the continuous presence of drug is required to sustain the reduction of XPO1 protein. Measurements by FCCS showed that brief exposure to selinexor (6 hours) resulted in rapid XPO1 occupancy, and drug washout caused sustained occupancy that was not relieved until 48 hours post-selinexor wash out. This relief of XPO1 occupancy by 48 hours after a 6 hour treatment of selinexor aligns with the recommended selinexor dosing schedule of BIW.

Target occupancy can be used to predict or optimize drug dosing schedules [22, 27]. Recent selinexor clinical publications have concluded that the RP2D for selinexor is 60 mg BIW [8, 9, 18]. The FCCS assay was used to measure XPO1 occupancy in tumors from mice treated *in vivo* with selinexor. This was the first time XPO1 target occupancy was measured in tumors and a dose-dependent effect of selinexor on complex formation was observed with >90% target saturation at 10 mg/kg selinexor in mice (∼50 mg flat dose in humans), a dose active against xenografts, and consistent with doses of selinexor that show anti-cancer activity in patients with heavily pretreated hematologic and solid tumors (10-15 mg/kg in mice is approximately equivalent to 50-70 mg flat dose in humans)[8, 18, 28]. Thus, the RP2D of 60 mg flat dose is within the range of lowest amount of selinexor (10 mg/kg) that reached the maximum target occupancy.

The FCCS assay was also used to confirm the selinexor clinical dosing schedule. Drug-target occupancy was measured over time post a single dose of selinexor in Z138 xenograft tumors from mice. The FCCS results showed that full occupancy occurred by 6 hours at all doses, but the duration of occupancy was dose-dependent. XPO1 occupancy was relieved by 24 hours at 5 and 10 mg/kg, 48 hours at 15 mg/kg, and 72 hours at 20 mg/kg selinexor. This finding supports the RP2D dosing regimen, where 10-15 mg/kg in mice (approximately equivalent to 50-75 mg flat dose in humans) is necessary to maintain XPO1 occupancy for up to 48 hours post-dose, thus requiring the twice weekly dosing for continuous target occupancy during the first half of each treatment week and washout / clearance during the second half of the week. The relief of target occupancy during the 5 day drug holiday is necessary for the recovery of normal cells, such as to allow CD8 T cells to launch an effective anti-tumor response [19], as well as to allow megakaryocyte differentiation to alleviate the main selinexor side effect, thrombocytopenia [29]. Together, these studies support the 60 mg BIW schedule empirically determined in the clinic [8, 9, 18].

Using an FCCS based assay developed to measure the dynamics of XPO1 target occupancy by selinexor, these studies support the selinexor RP2D dosing regimen for patients with heavily pretreated hematologic and solid tumors. Future studies are planned to measure XPO1 occupancy in patient tumor cells to investigate the correlation between the preclinical results described here to clinical occupancy and drug response in patients.

## MATERIALS AND METHODS

### Cell culture and reagents

The following cell lines (ATCC, except where noted) were grown in culture medium supplemented with 10% heat-inactivated fetal bovine serum (FBS, Gibco), 100 units/mL penicillin, 100 ug/mL streptomycin (Gibco), and1x GlutaMAX (Gibco) (except where noted), and maintained in a humidified incubator at 37°C in 5% CO_2_; Z138 (IMDM), THP-1 (RPMI), Kasumi-6 (RPMI, 2 mM L-glutamine, 1.5 g/L sodium biocarbonate, 4.5 g/L glucose, 10 mM HEPES, 1.0 mM sodium pyruvate, 2 ng/ml GM-CSF, 20% FBS), HEL (DSMZ, RPMI), MV-4-11 (IMDM), MM.1S (RPMI), HEK293 (EMEM), and NHEK (Lonza; KGM-Gold™ BulletKit™). PMBCs were isolated from human donor blood collected in Vacutainer® EDTA tubes (BD). PMBCs were separated by Ficoll (GE Healthcare) gradient.

The XPO1 SINE compounds selinexor and KPT-8602 were synthesized at Karyopharm Therapeutics Inc. Leptomycin B (LMB) was purchased from Cell Signaling. LMB was conjugated to the fluorophore Cy5 by solution phase synthesis as described in Patent WO2005117894A1. In brief, LMB (12.2 mg, 0.027 mmol, 1 eq), *N*-hydroxybenzotriazole (“HOBt,” 3.4 mg, 0.025 mmol, 1.1 eq), and (benzotriazol-l-yloxy)tripyrrolidinophosphonium hexafluoro- phosphate (PyBOP, 13 mg, 0.025 mmol, 1.1 eq) were dissolved in dry *N*,*N*- dimethylformamide (DMF, 400 μL). Amine A (0.025 mmol, 1.1 eq) and diisopropylethylamine (DIEA, 16 μL, 0.09 mmol, 4 eq) were subsequently added. The reaction was stirred at room temperature under nitrogen atmosphere for 20 hours. The reaction mixture was diluted with dichloromethane and washed with water, saturated sodium bicarbonate, and brine. The organic layer was dried over sodium sulfate, filtered, and concentrated in vacuo. The crude product (an oil) was applied to a silica flash column (0.5 × 5 cm) and eluted with 0% to 60% acetone/hexane. Fractions containing product were pooled and concentrated in vacuo.

### *In vitro* assays

Z138 cells in culture were treated with 1 μM selinexor either continuously for up to 32 hours or for 6 hours followed by drug removal and culture for up to 72 hours post-wash out. THP-1, Kasumi-6, HEL, MV-4-11, MM.1S and Z138 cells were treated in culture for 4 hours with either 0-10 μM selinexor, KPT-8602, or LMB. HEK293 cells were treated for 4 hours with 0, 0.01, 0.1, 1, or 10 μM selinexor. PBMCs and NHEK cells were grown without treatment. Lysates from cultured cells were prepared from confluent grown cell culture dishes. Cells were washed in PBS and lysed in 0.5 ml PBS with 0.04% Tween 20 and protease inhibitor on ice. Detergent concentration was reduced to 0.02% and lysates were cleared by ultracentrifugation (100,000 xg, 1 hour, 4°C). Supernatants were collected and directly subjected to FCCS analysis or stored at -80°C. Repeated freeze thaw cycles were performed on lysates prepared as described above by freezing at -80°C for several hours, thawing at room temperature and measuring drug target interactions after every cycle.

For the MTT cytotoxicity assay, THP-1, HEL, MV-4-11, MM.1S, and Z138 cells were seeded in 96-well flat bottom culture plates. Titrating concentrations of KPT-8602 were added to the wells and incubated for 72 hours at 37°C in a 5% humidified CO_2_ incubator. Triplicate wells per concentration were used to calculate IC_50_ curves. The CellTiter-Fluor Cell Viability Assay (Promega) was performed per the manufacturer's instructions. The assay was performed in triplicate. The inhibitory rate of cell growth was calculated using the formula: % growth inhibition = (1 – OD extract treated)/OD negative control x 100) [30].

### *In vivo* assays

Nude mice were inoculated in both flanks with Z138 cells. Once tumors reached approximately 250 mm^3^, mice were orally administered a single dose of 0, 5, 10, 15, or 20 mg/kg selinexor. Tumors were harvested at 6, 24, 48, 72, and 96 hours post-dose. Tumor samples were weighed and stored at -80°C until thawed on ice and transferred to a glass dounce homogenizer (1 ml). Bigger tumor tissue samples were cut in small pieces of 2 mm side length. Lysis buffer (200 μl of 10 mM PBS/150 mM NaCl, 0.5 mM MgCl_2_, 0.02% Tween-20, protease inhibitor) was added to each 100 mg tumor tissue and dounced on ice for 1 min. Homogenates were subjected to ultracentrifugation and cleared for 2 hours at 150,000 xg. Supernatant was transferred into an Eppendorf tube and centrifuged for another 15 min at 21,000 xg to separate the lipid layer. Lipid free supernatant was directly analyzed or stored at -80°C.

### Western blot

Protein lysates were analyzed by Western blotting to quantify the relative amount of XPO1 compared to GAPDH. Lysates from cultured cells or xenograft tissue was separated by SDS PAGE (10% polyacrylamide gel), electro blotted on nitrocellulose Hybond-ECL membranes and incubated with primary antibodies directed against XPO1 (Santa Cruz #sc-5505) and GAPDH as control (Hersteller). HRP signals of XPO1 and GAPDH bands were quantified by densitometry using ImageJ (NIH) software.

### FCCS analysis

FCCS measurements have been described in detail previously [20]. In brief, 2 overlapping laser beams illuminate a microscopic open detection spot in the test solution. Fluorescent molecules diffusing through the illuminated spot emit photons, which are recorded on highly sensitive avalanche photo detectors (APDs). Signal fluctuations caused by fluorescent molecules diffusing into and out of the detection volume carry information on diffusion time, molecular brightness and concentration. Samples with a volume of 20 μl were placed in glass bottom 384 well plates from SwissCI AG (6345 Neuheim, Switzerland) and FCCS measurements were performed either on a ConfoCor2 FCS unit connected to an Axiovert 100M equipped with a C-Apochromat 40x water immersion lens, NA 1.2 (Carl Zeiss, Jena, Germany) or an Insight plate reader (Evotec Technologies, Hamburg, Germany) fitted with a U-Apo300 40X water immersion lens, NA 1.15 (Olympus, Tokyo, Japan). Both systems used a 633 nm helium-neon laser for excitation of Cy5, AlexaFluor647 or DY647 dyes and the 488 nm laser line of an argon-ion laser to excite GFP. Fitting of the auto- and cross correlation functions and data analysis were carried out either in the ConfoCor2 software or with FCS+plus Analyze (Evotec technologies software). Data acquisition for samples in equilibrium took typically 20 – 60 s per sample.

Lysates of cells and tumor tissue were mixed with fluorescent tracer (LMB-Cy5) and fluorescent antibodies against XPO1 at concentrations of 20 nM and incubated for 20 min. Binding of LMB-Cy5 into the binding pocket of XPO1- antibody dimer forms a dually labeled complex, which can be quantified with great sensitivity. Occupation of binding sites on XPO1 prevents binding of LMB-Cy5 and such indicates target engagement by the test compound.

## SUPPLEMENTARY MATERIALS FIGURES AND TABLES



## Abbreviations

TSP: tumor suppressor protein
SINE: selective inhibitor of nuclear export
RP2D: recommended phase 2 dose
FCCS: fluorescence cross-correlation spectroscopy
FCS: fluorescence correlation spectroscopy
BIW: twice weekly
PBMC: peripheral blood mononuclear cell
LMB: leptomycin b
