# Supplementary material for: XPO1 target occupancy measurements confirm the selinexor recommended phase 2 dose
Source: Oncotarget. 2017 Nov 30;8(66):110503–16. doi: 10.18632/oncotarget.22801 (PMC5746399; doi:10.18632/oncotarget.22801)
Supplement: Supplementary file 1 [file oncotarget-08-110503-s001.pdf]

XPO1 target occupancy measurements confirm the selinexor recommended phase 2 dose

SUPPLEMENTARY MATERIALS

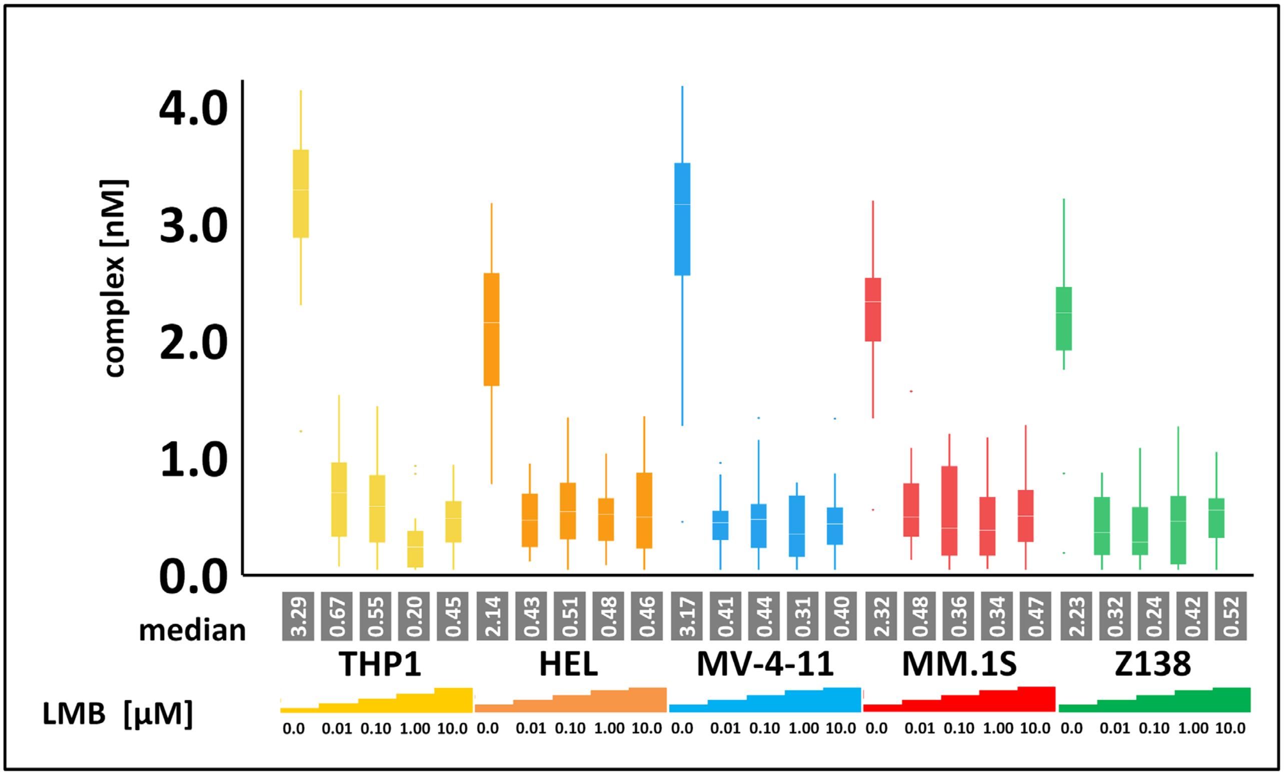

Supplementary Figure 1: LMB occupies XPO1 similarly in all cell lines at very low doses. FCCS measurements of XPO1 occupancy from THP-1, HEL, MV-4-11, MM.1S, and Z138 cell lines grown to confluency and treated with 0, 0.01, 0.1, 1, and 10 µM LMB for 4 hours. Cells were washed, harvested and lysed using PBS-Tween buffer and incubated with fluorescence labeled LMB (LMB_{647}) at a concentration of approximately 25 nM and ATTO488 labeled anti XPO1 antibody (XPO1 AB_{488}) for 2 hours and complex formation was analyzed on a ConfoCor2 Correlation Spectroscope. The normalized (untreated = 100%) values for the XPO1 complexes were plotted for LMB.
